# Poster Session I - A123 OBSTETRICS AND GYNECOLOGY HEALTHCARE UTILIZATION IN PERONS WITH IBS AND IBD

**DOI:** 10.1093/jcag/gwaf042.123

**Published:** 2026-02-13

**Authors:** P Shah, Z Nugent, C Bernstein, S R Shaffer

**Affiliations:** University of Manitoba Max Rady College of Medicine, Winnipeg, MB, Canada; University of Manitoba Max Rady College of Medicine, Winnipeg, MB, Canada; University of Manitoba Max Rady College of Medicine, Winnipeg, MB, Canada; University of Manitoba Max Rady College of Medicine, Winnipeg, MB, Canada

## Abstract

**Background:**

The prevalence of IBD in Canada in 2023 was approximately 320,000 Canadians, and nearly half are female. The prevalence of IBS was estimated to be 4.2% in based on the ROME IV criteria. Despite this, there is a paucity of data assessing healthcare utilization in persons with IBD and IBS especially at the time of menopause.

**Aims:**

To compare the age of onset of menarche and menopause in female patients with IBS, IBD and healthy controls as well as the healthcare utilization regarding care from obstetricians and gynecologists.

**Methods:**

Data were collected from questionnaires provided to persons in Manitoba with IBS and IBD, as well as from healthy controls as part of the IMAGINE study. Using administrative data from the Manitoba Centre for Health Policy, we identified healthcare utilization including visits to obstetricians/gynecologists. Controls were matched by age and sex (10:1) to each case of IBD or IBS. Questionnaires inquired as to ages of menarche, and the onset of menopause. Data were then analyzed with paired t-tests and non-parametric analysis.

**Results:**

A total of 441 female persons (285 with IBD, 124 with IBS, and 32 healthy controls) completed the survey. Of these, 96 (39.1%) had undergone menopause in the IBD group, 40 (32.3%)in the IBS group, and 14 (43.8%)in the healthy controls. The median age of menopause in the IBD group, IBS group and HC was 49, 48, and 50, (p = 0.24), respectively.) The median age of menarche was younger in the IBS (12 years) group compared to the IBD group (13 years) (p = 0.04).

From ages 15-70, persons with IBD had more encounters with an obstetrician/gynecologist than persons with IBS (p = 0.0009). Persons with IBD had more visits to an obstetrician/gynecologist when compared to matched controls (p < 0.0001), as did persons with IBS (p < 0.0001).

**Conclusions:**

There was no difference in age of menopause in our cohort, although persons with IBS had a younger age of menarche than IBD. Persons with IBD had more healthcare visits to a obstetrician/gynecologist than those with IBS, while both persons with IBD and IBS in our cohort had more visits compared to the general population. Further research is needed to understand why persons with IBD have an older age at menarche, and why persons with IBD and IBS seek more care from obstetricians/gynecologists.

**Funding Agencies:**

None

